# Local Drug Delivery Systems for Vital Pulp Therapy: A New Hope

**DOI:** 10.1155/2021/5584268

**Published:** 2021-09-15

**Authors:** Ardavan Parhizkar, Saeed Asgary

**Affiliations:** Iranian Centre for Endodontic Research, Research Institute for Dental Sciences, Shahid Beheshti University of Medical Sciences, Tehran 1983963113, Iran

## Abstract

Vital pulp therapy (VPT) is deliberated as an ultraconservative/minimally invasive approach for the conservation of vital pulpal tissues, preservation of dental structure, and maintenance of tooth function in the oral cavity. In VPT, following the exposure of the dental pulp, the environment is prepared for the possible healing and probable refunctionalisation of pulpal connective tissue. However, to succeed in VPT, specific biomaterials are used to cover and/or dress the exposed pulp, lower the inflammation, heal the dental pulp, provoke the remaining odontoblastic cells, and induce the formation of a hard tissue, i.e., the dentinal bridge. It can be assumed that if the employed biomaterial is transferred to the target site using a specially designed micro-/nanosized local drug delivery system (LDDS), the biomaterial would be placed in closer proximity to the connective tissue, may be released in a controlled and sustained pattern, could properly conserve the remaining dental pulp and might appropriately enhance hard-tissue formation. Furthermore, the loaded LDDS could help VPT modalities to be more ultraconservative and may minimise the manipulation of the tooth structure as well as pulpal tissue, which could, in turn, result in better VPT outcomes.

## 1. Introduction

Nowadays, vital pulp therapy (VPT) modalities are regarded as increasingly common ministrations and/or minimally invasive procedures [1] implemented to conserve/preserve the vitality of the dental pulp and maintain its function when the vital connective pulpal tissue has been compromised but not fully impaired by various stimuli [2, 3]. Currently, VPT is considered an important alternative ultraconservative approach to conventional root canal therapy techniques, since it seems to (i) promote regenerative endodontics [4], (ii) preserve dental structure and function in the oral cavity [5], (iii) cause more tooth resistance against masticatory forces compared to traditionally endodontically treated teeth [6], and (iv) maintain the physiological process in the exfoliation of primary teeth [7]. VPT modalities differ from minimally or semi-invasive (i.e., stepwise excavation, indirect pulp capping, and direct pulp capping) to relatively invasive (i.e. miniature, partial pulpotomy, and full pulpotomy) forms.

In brief, VPT is initiated with an access of the healthy layers of dental pulpal tissue following the removal of the inflamed pulp in different scales, from 1 mm in miniature pulpotomy to the entire tissue of the coronal chamber in full pulpotomy and haemorrhage control of the remaining vital pulp. After gaining haemostasis, the selected endodontic biomaterial is applied as close as possible to the surviving connective pulpal tissue and undifferentiated mesenchymal cells to conceivably induce cell differentiation and, subsequently, form of a hard tissue barrier [8, 9]. If the biomaterial is well adapted to the remaining vital tissue/surrounding dentinal walls in the absence of any blood clot and creates an impermeable seal, the healing process is expected to occur more rapidly with the most desirable outcomes [1]. In fact, VPT owes its success to the dental pulp, which is deliberated as a capable connective tissue with high potential for healing and regeneration [10, 11]. Existence of human dental pulp stem cells (DPSCs), grounds for cellular differentiation to odontoblasts, matrices for development of new tissues, and similar phenomena make the dental pulp a unique environment for regenerative processes [12]. Therefore, if the vital/surviving pulp is well protected from irritating agents and properly supported, it could maintain its vitality and resume its functions after injuries and inflammation. Consequently, specific endodontic biomaterials have been introduced for use in VPT to (i) dress and protect the pulpal tissue from its surroundings, (ii) assist the dental pulp in the preparation of a proper matrix for hard-tissue formation, and (iii) help the dental pulp regain its normal condition [13], which sequentially could result in less complicated or aggressive treatment procedures.

## 2. Local Drug Delivery Systems

Today, due to problems associated with systemic drug delivery methods, local drug delivery systems (LDDSs) have acquired great attention [14, 15]. Various studies have shown that LDDSs seem to be able to (i) transfer the loaded agent(s) to the target site and act as targeted drug delivery vehicles [16, 17], (ii) release the loaded agent(s), e.g., drugs/antibiotics/growth factors, in a designated, controlled, and sustained pattern [18], (iii) provide the anticipated therapeutic dose of agent(s) on the desired site [19], and (iv) lower the possible adverse effects of the systemic dose of the agent(s) [20]. LDDSs have long been used in medicine with acceptable degrees of success, and owing to the recent accomplishments in bio/nanotechnology, various types of LDDSs have started to be applied in different fields of dentistry from research to clinical applications and from operative dentistry to periodontology/periodontics [21, 22] and endodontology/endodontics [23].

Micro-/nanoparticles have been vastly studied for use in endodontic treatments [24]. A recent experimental/in vitro investigation has introduced an innovative local delivery system of bioceramic microparticles, made from hydroxyapatite and *β*-tricalcium phosphate, coated with a biopolymer, i.e., polylactic coglycolic acid (PLGA), so as to transfer loaded antibiotics to the root canal system (target drug delivery) and remove dominant intracanal microorganisms, exhibiting successful outcomes [25]. Comparably, Virlan et al. have presented PLGA microparticles with added zein loaded with an antibiotic (i.e., amoxicillin) and PLGA microparticles in a PLGA/calcium phosphate cement loaded with growth factors to be used in root canal treatments and endodontic regeneration, respectively [26]. Seemingly, PLGA microparticles have been/are immensely regarded as appropriate microcarriers for sustained drug release by different investigations [27–29]. Similarly, Álvarez et al. have suggested microencapsulation techniques as well as the application of microparticles in vitro/in vivo for the delivery of different agents (i.e., antibiotics, drugs, and growth factors) to the root canal cavity [30]. Moreover, analogous in vitro/in vivo studies have shown successful results when loaded/seeded LDDS are employed in endodontic ministrations, e.g., the addition of amoxicillin-loaded microspheres to mineral trioxide aggregate and the delivery of antibiotic-loaded microcarriers via the calcium silicate-based cement to the anticipated site [31] as well as the application of alginate/laponite hydrogel biopolymer-based microspheres transferring DPSCs and vascular endothelial growth factor (VEGF) as a signalling protein for regenerative endodontics to the target space [32]. A recent study claims that microspheres, hydrogels, and fibers are the most common vehicles for the transfer and sustained release of agents in endodontics [33].

Furthermore, “Gels” have been enormously used in dentistry as a local drug delivery system, especially in periodontics [34–36], and due to their promising potentials and capabilities, they have been investigated in endodontics [24, 37]. Hydrogels with their cross-linked hydrophilic network have shown comparable properties to the dental pulpal tissue [38] as well as the ability to carry, transfer, and release agents/drugs to the intended site. Consequently, they are being regarded as suitable vehicles for the regeneration of the dentine-pulp complex and corresponding protocols [39]. Moreover, it has been demonstrated that hydrogels can be a very useful structure for controlling the porosity and viscosity of endodontic intracanal scaffolds [40]. Besides, injectable hydrogels merged with stem/progenitor cells seem to be considered a favourable technique/method for possible regeneration of the dentine-pulp complex and could play an important part in endodontic regeneration protocols [41]. Natural polymer-based hydrogels have shown bioactivity, high biocompatibility, and biodegradability by hydrolysis/enzymes [42] whereas synthetic polymer-based hydrogels have presented acceptable mechanical properties, reliable durability, and appropriate thermostability compared to natural hydrogels [43]. Therefore, the hybrid form of hydrogels could be identified for use in endodontic regeneration and engineering of pulpal tissue, exhibiting both characteristics and properties of their components [44]. In addition, Ribeiro et al. have recently introduced an injectable matrix metalloproteinase- (MMP-) responsive nanotube-modified gelatine hydrogel for possible sustained intracanal application to be used against endodontic infections owing to its credible affinity of cell tissue and degradability [45]. Nevertheless, hydrogels and their application in endodontic treatments should be further investigated.

Films, strips, and fibers have been widely studied for oral drug delivery, particularly in the management of periodontal diseases and endodontic problems [30, 46]. Chitosan/PLGA and degradable PLGA films have been introduced to the periodontal pocket as local drug delivery systems to transfer and/or release loaded agent(s)/drug(s), e.g., ipriflavone, and act against periodontal pathogens [47, 48]. In addition to films, biodegradable or nonbiodegradable polymer strips have been reported to be an efficient method of administering antibacterials for periodontal treatments [49]. Drug-loaded strips, specifically with tetracycline hydrochloride and metronidazole, have been successfully used in patients with advanced periodontal pathosis, with tetracycline being more successful than metronidazole in the conducted investigations [50]. Moreover, strips have been used as mucoadhesive local delivery systems for fluoride-incorporated layered double hydroxides in periodontal treatments with reasonable safety and efficient release of fluoride ions to prevent caries through an ion-exchange mechanism with the surrounding environment [51]. Similarly, fibers have shown significant potential for the transfer of agents/bioactive materials/drugs, e.g., tetracycline [52], to their designated target sites (e.g., periodontal pocket) and releasing them in a controlled pattern [53]. Moioli et al. claim that nanofibers, along with microparticles and hydrogels, can be considered for use in the regeneration of tooth and periodontal tissues [54], with a notable focus on the PLGA micro-/nanofiber system, a biopolymer approved for numerous applications and production of scaffolds/matrices in tissue engineering [55]. Due to the reported biocompatibility and biodegradability, polymer-based nanofiber systems made from natural or synthetic biomaterials seem to be effective means for local delivery [21]. Sequentially, fibers have been deliberated for use in endodontic treatments/investigations. Haugen et al. claim that nanofibers can be considered for pulp tissue engineering and regenerative endodontics due to their potential use for dental tissues [56]. Related studies have reported that fibers can act biomimetically to create microenvironments and behave as an appropriate scaffold for the pulp-dentine complex, mimicking the native pulp and function [57]. Fibers and nanofibrous constructs can be regarded as an alternative approach for intracanal disinfection [58], since they appear to operate/serve as a drug delivery vehicle for a number of antibiotics, revealing analogous antibacterial effects to antibiotic pastes, however, with minimum concentration of the drugs used [59]. It has been shown that chitosan/gelatine nanofiber membranes are able to carry cinnamon extract and sustained release the loaded substance to prevent infection on the implanted site [60]. Polymeric fibers incorporated with curcumin have shown antibacterial properties and possible use for the disinfection of root canal cavity [61]. Moreover, clindamycin-modified triple antibiotic polydioxanone-polymer-based nanofibers have shown to act effectively against intracanal microbiota and different root canal microorganisms [62].

Additionally, LDDSs have shown to be able to transfer a variety of agents to their target sites, including calcium hydroxide (CH) [63] as the first “Gold Standard” biomaterial for VPT [64] and triple antibiotic paste (TAP) and/or modified TAP (mTAP), to combat microorganisms and bacterial biofilm [65]. A recent study has shown that calcium hydroxide-loaded PLGA nanoparticles as LDDSs can show a prolonged profile of agent/drug release and excellent penetration in the dentinal tubules compared to CH alone [66]. Furthermore, LDDSs could show other characteristics beside their ability to carry/transfer agents owing to the types of (bio)materials used in their synthesis. Bioceramics, e.g., silica, calcium phosphates, and calcium silicates, have been widely used in bone healing [67]. Moreover, ceramic microparticles/scaffolds made from hydroxyapatite and bi/tricalcium phosphates have shown biocompatibility, bioactivity, and ability to form hard-tissue barriers [68]. Since the existence of a dentinal construct is of utmost importance in VPT [69], bioceramic LDDSs could be a noted candidate for use in VPT modalities, specifically calcium silicate-based cements. Besides, polymer-based LDDSs have revealed an ability to control/sustain-release therapeutic agents as well as carry hydrophobic and hydrophilic drugs [70]; e.g., biocompatible and biodegradable hydrogels have shown to be able to release Ca^2+^ from the loaded calcium hydroxide [63]. Inorganic materials and synthetic/natural polymers have been advocated to devise LDDSs for dentin-pulp tissue engineering [71], since (i) bioactive molecules (BMs) needed for regeneration could be encapsulated and transferred by the mentioned LDDSs, (ii) BMs could be released in the concentrations needed for tissue engineering, and (iii) LDDSs can function as fundamental scaffolds and provide support for the ingrowth of the tissue during regeneration [67].

## 3. Biomaterials

It is believed that dental biomaterials have revolutionised/caused present-day approaches and treatments in dentistry, specifically in periodontics and endodontics [72]. Vital pulp therapy, as a modern concept and accepted modality [73], is grounded on the removal of causative factors which results in the reduction/elimination of inflammation and pathosis of the dental pulp, stimulation of the connective tissue to rebuild itself, and proper haemodynamics in the pulpal tissue [73, 74]. Studies have reported that an effective VPT needs alleviation of further injury to the remaining odontoblasts and encouragement of new odontoblasts to differentiate [75]. Consequently, the applied biomaterials considered for VPT should help to achieve sealing of the exposure site, reduction of inflammation, formation of a dentinal bridge, and differentiation of potent cells [76]. Furthermore, they should be ideally biocompatible, bioactive, bioinductive, and bioconductive and be able to adhere to the tooth structure, preserve sealability, display insolubility, demonstrate dimensional stability, and show nontoxicity [69]. Studies and investigations have focused on different biomaterials for VPT; however, the most important/common ones are based on calcium hydroxide (e.g., Dycal) and calcium silicate (e.g., mineral trioxide aggregate “MTA,” calcium enriched mixture “CEM” cement, and Biodentine). The biomaterials are applied after VPT initial clinical stages and following the necessary primary preparation of the exposure site and pulpal tissue.

### 3.1. Calcium Hydroxide (CH)

Calcium hydroxide is considered the traditional biomaterial for pulp capping and was introduced to dentistry by Hermann et al. in the 1930s [77]. Once the “gold standard” for VPT [11], CH is able to release/dissociate calcium and hydroxyl bioactive ions to its surrounding environment [78], trigger wound healing, and promote regeneration in pulp capping procedures [12]. It seems that the velocity of ionic dissociation of calcium hydroxide greatly depends on/is defined by its vehicle [77].

Calcium hydroxide has been compared with adhesives and bonding systems for use in VPT. It is expected that adhesives are able to effectively seal the exposed pulp and stop the leakage of microorganisms into the connective tissue. However, it has been shown that the exudation and moisture from the exposure site can affect the seal caused by adhesives/bonding systems and bacteria may leak into the pulp [79]. In addition, the potential cytotoxicity of adhesives and the needed techniques for their application have questioned their solicitation in VPT [80, 81], and as a result, using a bonding system next to CH is not currently recommended [5]. In comparison with resin modified glass ionomer (RMGI) materials, CH has shown to be the material for VPT, since RMGI can cause moderate to severe pulp inflammation and possible tissue necrosis with lack of dentinal bridge formation [82].

Although calcium hydroxide has exhibited basic pH values [83] and could be theoretically regarded as an antibacterial agent, application of CH has resulted in poor antibacterial activity as well as lack of trusted seal, no adherence to dentinal walls [11], and a porous dentinal bridge [84]. Therefore, CH is no longer elaborated as the material of choice for VPT. Several calcium silicate-based cements/biomaterials have been proposed for use as replacements for CH, since they have revealed higher rates of success amongst different biomaterials used for VPT. Calcium silicate-based materials have shown to release high amounts of calcium and, thus, should be able to induce hard-tissue (i.e., dentinal bridge) formation [85]. In a recent systematic review, it has been claimed that MTA, CEM cement, and Biodentine are the common calcium silicate-based cements of choice to be used in VPT modalities for different conditions of the pulp [86].

### 3.2. Mineral Trioxide Aggregate (MTA)

Various studies state that MTA is a more successful calcium silicate-based endodontic cement/biomaterial in comparison to CH, and thus, MTA has claimed the place of CH in VPT [87, 88]. Mineral trioxide aggregate is a fast setting bioactive calcium-silicate based cement [89], which means it can release calcium ions and form an interfacial layer between the biomaterial and dentine. Consequently, MTA has shown to cause biomineralisation beneath the pulp exposure site and has been advocated to maintain the vitality of pulpal tissue [90]. The release of calcium ions from MTA and their reaction with phosphates in tissue fluids cause the cement to be capable of developing the formation of hydroxyapatite in contact with tissue body fluids [91] as well as the induction of the regenerative process through forming nanocrystals [92]. A histological study has indicated that MTA is effective on the regeneration potential of the dental pulp tissue and can cause adequate metabolic activity and favourable cellular response, resulting in less tunnel effect and higher clinical success rate [93, 94]. Therefore, there have been a number of expanding applications for MTA, e.g., direct [95]/indirect pulp capping next to the repair of root/furcation perforations [96] and apexification [97], making MTA superior calcium silicate-based cement over CH [98]. Although MTA has shown excellent properties and acceptable biocompatibility, there are drawbacks to MTA, including high price, long setting time, triggering discolouration [99], and difficult manipulation/handling [100, 101]. Nonetheless, MTA is currently regarded as the “Gold Standard” calcium silicate-based cement/biomaterial for VPT modalities [102].

### 3.3. Biodentine™ (BD)

Biodentine™ is a tricalcium silicate-based biomaterial with properties appropriate for endodontic treatments, particularly VPT. Biodentine™ has revealed acceptable marginal seal due to its mechanical retention in dentinal tubules and adequate bond strength to dentine comparable to that of MTA [103]. In addition, when BD is used as calcium silicate-based cement for direct pulp capping and/or pulpotomy in mature permanent teeth with carious exposure, it has shown similar success rates to MTA [90]. Furthermore, studies have demonstrated that once BD is directly applied onto the dental pulp, it can (i) induce reparative dentine comparable to primary dentine [104], (ii) reliably form a dental bridge [105], and (iii) cause possible decrease/absence of inflammatory response in the pulp as well as probable increase in cell differentiation [106]. Moreover, Biodentine has exhibited improvements in properties in comparison to other calcium silicate-based cement, i.e., high compressive strength, short setting time [107], and favourable bond strength. These features have resulted in a wider scope of use in endodontic therapy, especially VPT modalities, e.g., in full pulpotomy for the management of irreversible pulpitis with/without apical periodontitis [108, 109]. Additionally, Biodentine has revealed less solubility, tighter seal, and higher mechanical strength when compared to CH [110]. Despite excellent properties, BD has shown to lose its radiopacity with time which makes it difficult to be radiographically observed in long term [90].

### 3.4. Calcium-Enriched Mixture (CEM) Cement

Calcium-enriched mixture cement, as a calcium silicate-based biomaterial, has shown to have favourable physical properties, i.e., flow, film thickness, primary setting time [111], and comparable clinical applications to MTA, e.g., in the repair of root resorption/furcation perforation and use in VPT modalities [112]. Various studies have shown that CEM cement exhibits acceptable biological properties, has proved to be biocompatible, and is able to release calcium ions during its setting reaction showing bioactive properties. It seems that the reaction between calcium ions and phosphorus results in the formation of hydroxyapatite; which, in turn, could cause enzyme activity in cells, preparing a matrix for healing and possible hard-tissue formation [113]. Besides, the antibacterial activity of the cement is similar to that of CH and appears to be superior to that of MTA [112, 114]. Other studies have claimed that CEM cement has acceptable sealability comparable to that of MTA after root resection and in apical sealing [115]. In addition, there seems to be no statistical difference in the sealing ability between CEM cement and MTA in the apical area, which can be related to the chemical properties and handling characteristics of CEM cement [116]. CEM cement is claimed to be suitable calcium silicate-based cement for use in a variety of clinical applications, e.g., VPT modalities, root-end filling, perforation, resorption, and regenerative endodontics [112].

### 3.5. Bioactive Glass (BG)

Bioactive glass is a bioceramic material consisting of silica, calcium oxide, sodium oxide, and phosphorus pentoxide and has been regarded as an active biomaterial for use in hard-tissue formation through the development of the hydroxyapatite layer [117, 118]. Therefore, it can be hypothesised that BG can be considered for use in dental treatments when formation of hydroxyapatite and biocompatibility without cytotoxic effects are necessary for the achievement of a successful outcome [119]. Subsequently, a bioactive glass-based endodontic sealer has been introduced and further efforts are on the way to enhance glass-based bioactive cement [69]. Consequently, BG seems to be a promising/potential biomaterial for use in VPT modalities, so as to cause and/or induce hard-tissue formation, i.e., a dentinal bridge, on the pulp exposure site if applied as a pulp capping biomaterial directly over the pulp [120]. Further investigations are, nevertheless, necessary for the application of BG as a dressing in VPT.

It has been shown that LDDSs can have efficient impacts on the outcomes of VPT, since they are likely to be able to (i) transfer/deliver antimicrobials directly to the exposure site, (ii) remove the pathogenic microbiota, (iii) regulate/reduce the inflammation, and (iv) decrease the destruction in the pulpal tissue, as were reported by Kalyan et al. using chlorhexidine polymer scaffold as LDDSs for VPT [121]. In addition, the local regeneration of the dentine-pulp complex has been suggested/investigated using a local delivery system loaded with perfect combination of growth factors, e.g., fibroblast growth factors and bone morphogenetic proteins, with a scaffold for DPSCs [69, 122, 123]. Moreover, use of a biostimulation membrane in the dentine-pulp complex has shown success in the maintenance of dental pulp tissue as well as the physiological process of exfoliation in primary teeth [2]. It has been suggested that, with the application of local biodegradable/biocompatible carrier vehicles to transfer signalling/bioactive molecules to the capping site in VPT, induction of odontoblastic-like cell differentiation, formation of reparative dentine, and stimulation of fibrodentine may be anticipated, the phenomena which, in turn, could result in the reconstitution of normal tissue architecture in the dentine-pulp interface [124]. A comparable experimental study has shown that chitosan/*β*-glycerophosphate hydrogel has been able to sustain-release VEGF and may trigger the odontogenic differentiation of DPSCs, promoting/contributing to the idea of using LDDSs in VPT modalities [125].

Currently, local delivery systems are used to transferring drugs/agents to their target sites so that they could be released in their desired/therapeutic concentration and optimum time, resulting in their needed effect. In VPT, vitality of the remaining tissue and maintenance of its capacity for healing is of great importance. Consequently, direct transfer of drugs/agents, i.e., biomaterials in VPT, to the area of pulp exposure, individually or as a combination, on a specially designed vehicle may prepare the environment for healing of the exposed site as well as the pulpal tissue. The direct transfer and subsequent release of the loaded biomaterials on the pulpal wound in the required dose and controlled manner next to further cell differentiation and less inflammation of the remaining pulp are anticipated to be observed in the mentioned strategy/method. Furthermore, the local delivery of biomaterials could compensate more for the extension of the exposure site and manipulation of the remaining pulp, making the technique more ultraconservative. Hence, with the proper choice of biomaterials selected for the local delivery, the promoted induction/formation of hard tissue, i.e., a dentinal bridge, is anticipated.

## 4. Conclusions

The agents/biomaterials needed for VPT could be locally transferred to the target site, i.e., pulp exposure, using small-sized local delivery systems. This allows specific controlled release of loaded biomaterials/molecules/substances/drugs to the desired location while enhancing the effect(s) of the agents. In the local delivery, the transferred agents will be in close proximity to the pulpal connective tissue and could further push the differentiation of DPSCs to odontoblasts. In addition, the preparation of pulp exposure site shall be reduced to its minimum/smallest possible size, which is of great significance in ultraconservative VPT approaches. Moreover, and although local dentine regeneration seems a difficult task, due to the type of biomaterials used for the synthesis of the delivery system and the kind of vehicle employed for agent/drug transfer, the formation of dentinal bridge could be more encouraged and induced.

Despite the low level of evidence on the application of LLDSs in VPT, next to local transfer of biomaterials to the pulpal exposure site via microcarriers/vehicles, it seems that LDDSs should be practised in VPT in experimental/in vitro and in vivo investigations next to animal models as well as related randomised controlled trials after being properly produced, perfectly experimented, vigilantly tested, and carefully evaluated. Patients' signs and symptoms should be recorded, clinical examinations should be wisely performed, and radiographic evaluations ought to be attentively considered. In addition, the formation of a dentinal bridge and the healing of pulpal tissue should be evaluated, analysed, and valued.
